# Supplementary material for: Identification of Novel Fusion Genes in Bone and Soft Tissue Sarcoma and Their Implication in the Generation of a Mouse Model
Source: Cancers (Basel). 2020 Aug 19;12(9):2345. doi: 10.3390/cancers12092345 (PMC7565474; doi:10.3390/cancers12092345)
Supplement: Supplementary file 1 [file cancers-12-02345-s001.zip › cancers-871079-supplementary conversion/cancers-871079 supplementary figure.pdf]

Supplementary material

Identification of Novel Fusion Genes in Bone and Soft Tissue Sarcoma and Their Implication in the Generation of a Mouse Model

Yasuyo Teramura, Miwa Tanaka, Yukari Yamazaki, Kyoko Yamashita, Yutaka Takazawa, Keisuke Ae, Seiichi Matsumoto, Takayuki Nakayama, Takao Kaneko, Yoshiro Musha and Takuro Nakamura

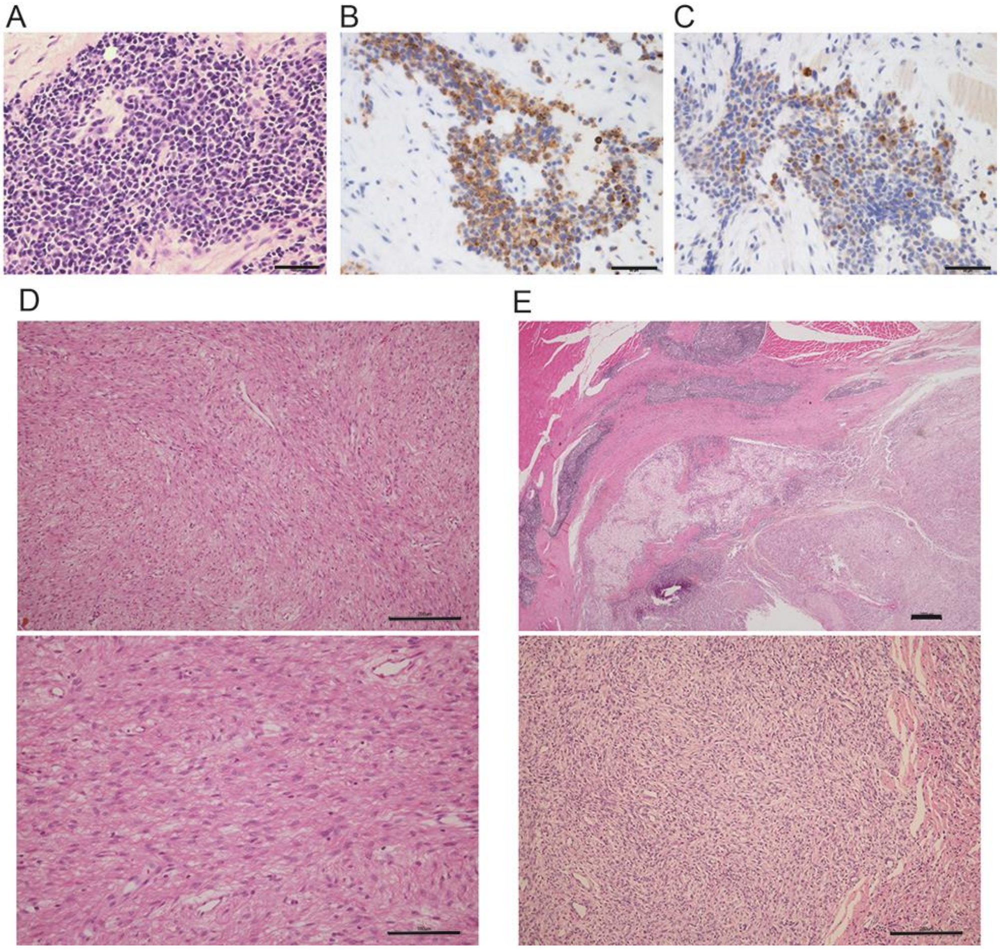

Figure S1. Histological findings of *EWSR1-ATF1-*, *GAB1-ABL1-*, and *EWSR1-CREB1*-positive cases. **(A–C)** Case 1 with *EWSR1-ATF1*. Although tumor cells show round cell morphology by H&E staining **(A)**, they are positive for melanocyte markers HMB45 **(B)** and Melan A **(C)**. Scale bars: 50 μm. **(D)** Case 31 with *GAB1-ABL1*, showing paucicellular proliferation of spindle tumor cells dispersed in fibrous matrix. Scale bars: 200 μm (top) and 100 μm (bottom). **(E)** Case 33 with *EWSR1-CREB1*, showing tumor nodule surrounded by fibrous capsule with lymphoplasmacytic infiltration (top, scale bar: 400 μm). Tumor cells contains moderate amount of cytoplasm with round nuclei (bottom, scale bar: 200 μm).

© 2020 by the authors. Licensee MDPI, Basel, Switzerland. This article is an open access article distributed under the terms and conditions of the Creative Commons Attribution (CC BY) license (http://creativecommons.org/licenses/by/4.0/).
