# Supplementary figures and images for: Identification of Novel Fusion Genes in Bone and Soft Tissue Sarcoma and Their Implication in the Generation of a Mouse Model
Source: Cancers (Basel). 2020 Aug 19;12(9):2345. doi: 10.3390/cancers12092345 (PMC7565474; doi:10.3390/cancers12092345)

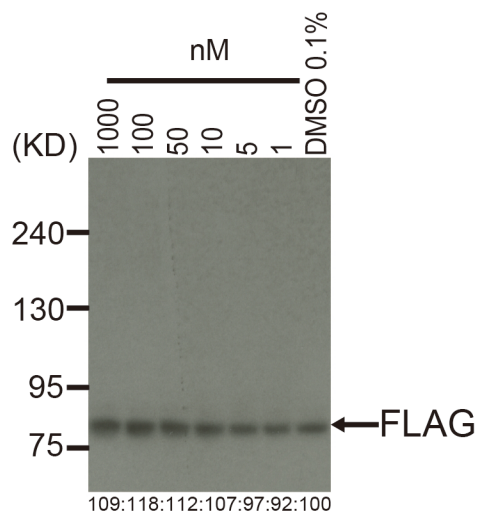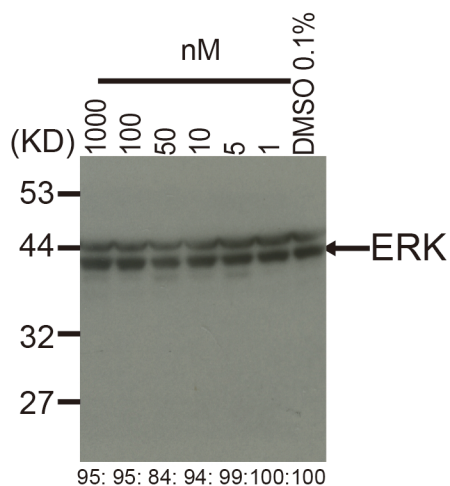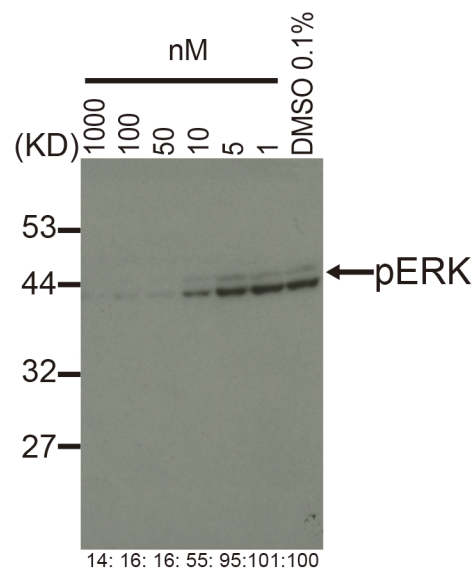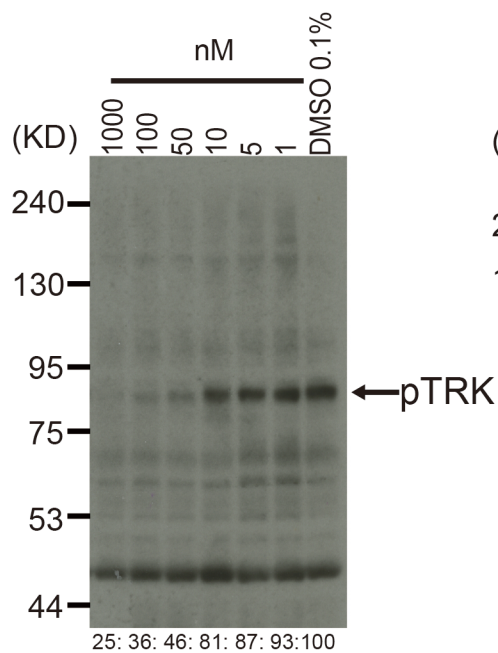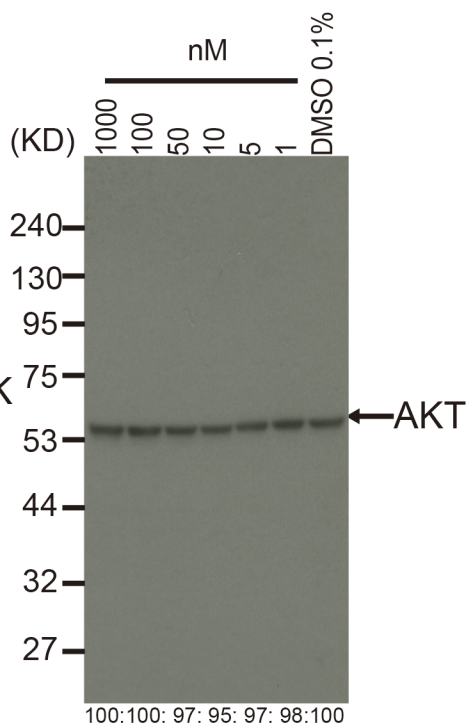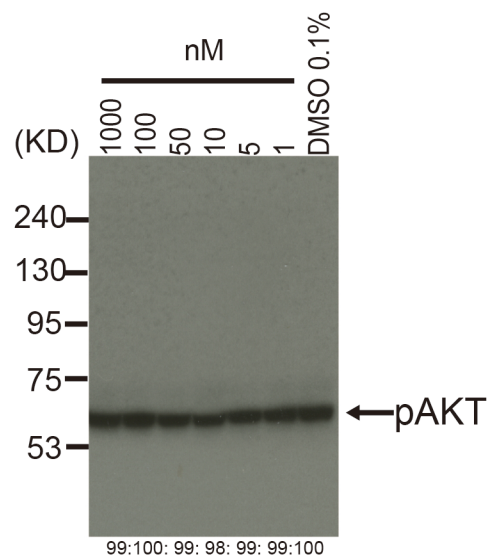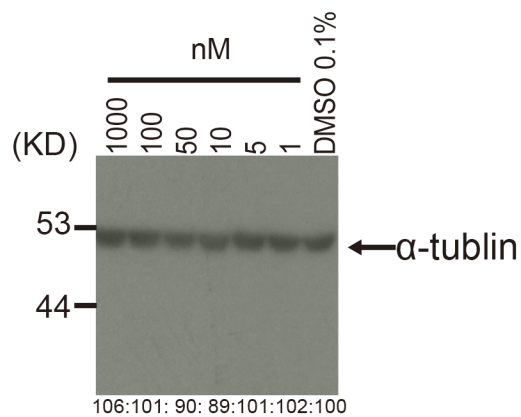

Supplement: Supplementary file 1 [file cancers-12-02345-s001.zip › cancers-871079-supplementary conversion/cancers-871079-Figure S2-western blot.pdf]
